# Role of Gangliosides in Peripheral Pain Mechanisms

**DOI:** 10.3390/ijms21031005

**Published:** 2020-02-03

**Authors:** Péter Sántha, Ildikó Dobos, Gyöngyi Kis, Gábor Jancsó

**Affiliations:** Department of Physiology, University of Szeged, Dóm tér 10, H-6720 Szeged, Hungary; santha.peter@med.u-szeged.hu (P.S.); kisne.dobos.ildiko@med.u-szeged.hu (I.D.); karcsune.kis.gyongyi@med.u-szeged.hu (G.K.)

**Keywords:** pain, ganglioside, primary sensory neuron, spinal cord, TRPV1, nerve injury, lipid raft, B subunit of cholera toxin

## Abstract

Gangliosides are abundantly occurring sialylated glycosphingolipids serving diverse functions in the nervous system. Membrane-localized gangliosides are important components of lipid microdomains (rafts) which determine the distribution of and the interaction among specific membrane proteins. Different classes of gangliosides are expressed in nociceptive primary sensory neurons involved in the transmission of nerve impulses evoked by noxious mechanical, thermal, and chemical stimuli. Gangliosides, in particular GM1, have been shown to participate in the regulation of the function of ion channels, such as transient receptor potential vanilloid type 1 (TRPV1), a molecular integrator of noxious stimuli of distinct nature. Gangliosides may influence nociceptive functions through their association with lipid rafts participating in the organization of functional assemblies of specific nociceptive ion channels with neurotrophins, membrane receptors, and intracellular signaling pathways. Genetic and experimentally induced alterations in the expression and/or metabolism of distinct ganglioside species are involved in pathologies associated with nerve injuries, neuropathic, and inflammatory pain in both men and animals. Genetic and/or pharmacological manipulation of neuronal ganglioside expression, metabolism, and action may offer a novel approach to understanding and management of pain.

## 1. Introduction

Gangliosides are abundantly occurring sialylated glycosphingolipid s serving diverse functions in the nervous system. These glycosphingolipids are primarily membrane-localized and through their oligosaccharide portion constitute specific recognition sites interacting with a variety of extracellular agents [1]. Gangliosides are integral components of membrane lipid microdomains (rafts) and may interact with membrane-bound functional proteins influencing their activation. Association of membrane receptor molecules with lipid microdomains has been shown to participate in the activation of a variety of ion channels or neurotransmitter receptors, including transient receptor potential (TRP) channels [2,3,4]. Gangliosides are also critically implicated in the mediation of the cellular actions of neurotrophic molecules, such as nerve growth factor and glial-derived neurotrophic factor [2,5]. Hence, gangliosides are important regulators/modulators of neural processes encompassing neural development and regeneration, intercellular communication, neurotrophic action, neurotransmission and other receptor-mediated functions [6,7,8,9,10]. Gangliosides are expressed in primary sensory neurons, in part in a function-dependent manner, which makes gangliosides possible molecular targets of pharmacological manipulations of pain-sensing neurons (vide infra). The present review attempts to summarize available evidence supporting the role of gangliosides in conveying sensory, in particular nociceptive information from the periphery towards the central nervous system by primary sensory neurons under physiological and pathological conditions.

## 2. Distribution of Gangliosides in Sensory Ganglia and Spinal Cord

Gangliosides are sialylated glycosphingolipid components of the outer leaflet of all animal cell membranes particularly abundant in the nervous system. Gangliosides are derived from ceramide, the condensation product of by condensation of sphingosine and a long chain fatty acid. The decisive step of the ganglioside biosynthesis is catalyzed by glucosylceramid synthase (GCS) also denoted as UGCG (UDP-glucose ceramide glycosyltransferase). Glucosylceramide is further conjugated with galactose producing lactosylceramid which is further conjugated with carbohydrates to form sialylated glycosphingolipids, traditionally denoted as gangliosides, or globosides, also called neutral (asialo) glycosphingolipids. Gangliosides are synthesized by conjugation by sialyltransferase of sialic acid (N-acetyl-neuraminic acid) to the galactose end of the lactosylceramide molecule creating GM3 ganglioside. Different ganglioside species are synthesized by further conjugation of carbohydrates and sialic acid in the oligosaccharide chain. The widely used nomenclature to classify different types of ganglioside species relies on the thin-layer chromatography-based separation introduced by Svennerholm [11]. Letter G refers to ganglioside, the second letter indicates the grade of sialylation (mono-, di- and trisialic gangliosides); the number refers to the order of migration of the ganglioside on thin-layer chromatography. In the nervous system the most abundant gangliosides are GM1, GD1a, GD1b, and GT1b. Catabolism of gangliosides is catalyzed by neuraminidase (N-acetyl-α-neuraminidase, Neu3) which hydrolyses sialic acid residues; carbohydrate moieties are removed by sequential hydrolysis of the neutral tetrasacharide chain by β-galactosidase, β-hexosaminidase, lactosylceramide-β-galactosidase, and glucosylceramidase. The key step of the degradation of globoseries glycosphingolipids is catalyzed by α-galactosidase. While genetic mutations causing dysfunction of the enzymes of ganglioside synthesis are usually lethal, mutations affecting the enzymes of ganglioside or globoside degradations lead to the development of different forms of lysosomal storage diseases. These rare conditions are characterized by the accumulation of gangliosides or intermediary molecules of the ganglioside degradation.

The possible role of gangliosides in organ and, in particular, neuronal functions has been studied in mice with genetic deletion of the GCS gene responsible for the expression of the key enzyme of ganglioside synthesis. Systemic disruption of the GCS gene resulted in the absence of all glucosylceramide-based glycosphingolipids and early embryonic lethality [12]. Mice with targeted deletion of the GCS gene in neurons were born normal but developed nervous dysfunctions shortly after birth and died within three weeks after birth. Altered neuronal morphology, impaired neurite outgrowth and disturbed myelinization of peripheral axons were demonstrated in these animals [13].

Early studies on the localization of gangliosides in sensory ganglia used the highly specific binding of choleratoxin B subunit (choleragenoid, CTB) and its conjugates, such as CTB-horse radish peroxidase (CTB-HRP) to the GM1 ganglioside [14,15,16]. It has been demonstrated that a morphologically well-defined population of primary sensory neurons, the large light, neurofilament rich neurons specifically bind CTB, whereas small dark neurons do not exhibit CTB binding [17]. Functionally, most large light neurons convey mechanoreceptive information, while small dark neurons are mostly nociceptive [18].

The demonstration of the distribution of gangliosides in the nervous system and, in particular, in primary sensory neurons and the spinal cord was significantly advanced by the production of highly specific antibodies against distinct ganglioside species [19,20,21]. Of the four major gangliosides, GM1, GD1a, GD1b, and GT1b which comprise the majority of total brain gangliosides of the mammalian brain, GD1b is expressed in both the white and the gray matter of the spinal cord, whereas GT1b is moderately expressed in all Rexed’s laminae of the gray matter [21]. Other gangliosides are limited to more specific areas. The findings that immunohistochemistry using antibodies recognizing GD1a revealed a selective staining of Rexed’s laminae I and II are of particular interest, since these laminae are the major termination sites of C- and Aδ-fiber nociceptive afferents [18,22,23]. In support of a possible role of GD1a ganglioside in the function of nociceptive primary afferent neurons, immunohistochemistry revealed that 90% and 18% of small and medium-sized neurons, respectively, and only 11% of large neurons were stained in dorsal root ganglia with antibodies against GD1a [20]. A close correlation between ganglion cell size, conduction velocity, and afferent fiber function is well established; smaller neurons possess C- and Aδ-fibers and are mostly nociceptive in function [24].

Interestingly, ganglioside GM1 could not be demonstrated in the spinal cord dorsal horn by GM1 immunohistochemistry [21]. However, the lack of immunoreactivity to a ganglioside epitope does not prove that the ganglioside is absent, since ganglioside immunoreactivity depends on several factors, including the density of the ganglioside in the membrane and the ceramide portion of the ganglioside [21]. Indeed, the presence of ganglioside GM1 was proved by making use of the highly specific binding of CTB to that ganglioside species. CTB binding was observed in the deeper layers of the spinal dorsal horn and large neurons in sensory ganglia [25]. It is important to note that this pattern of distribution of ganglioside species is characteristic of intact rats and mice [19,21]. Injuries of mechanical or chemical nature can profoundly alter the neural expression of specific gangliosides (vide infra).

## 3. Human Diseases Affecting Glycosphingolipid Metabolism and Pain

Much has been learned of the role of glycosphingolipids in pain mechanisms from investigations into the pathobiochemistry of human diseases affecting glycosphingolipid /ganglioside metabolism. Fabry or Anderson-Fabry disease is an X-chromosome-linked hereditary lipid storage disease caused by various mutations of the human GLA gene resulting in a decreased expression and activity of the lysosomal α-galactosidase A enzyme in the cells including neurons and glial cells of the peripheral nervous system. Reduced enzymatic activity affects the degradation of the complex glycosphingolipid globotriaosylceramide (Gb3). Prevailing consequence of the impaired catabolism of Gb3 is peripheral neuropathy affecting neurons of autonomic ganglia and Aδ- and C-fiber sensory ganglion neurons [26]. Gb3-induced small fiber neuropathy is characterized by an early onset of pathological pain sensations including intermittent burning pain attacks (Fabry attacks), impaired thermosensation (initially reduction of cold sensation) and acroparaesthesia. It must be mentioned that sensory symptoms change during the pathology: many early manifestations suggest a hyperactivity of nociceptive and other sensory neurons, while later in life deficits of the sensory functions are dominant. This time course was also observed in genetically modified animal models showing the characteristic phenotype of the disease [27]. Symptoms of autonomic neuropathy such as reduced sweating and hypohydrosis, reduced heart rate variability, orthostatic hypotonia and gastrointestinal dysmobility are also frequent complications of the disease [26]. In different animal models, similar abnormalities of sensory and autonomic functions have been observed after genetic deletion of the α-galactosidase gene [28,29,30]. Prominent morphological changes of the affected thin myelinated and unmyelinated sensory axons are the reduction of the intraepidermal fiber density in the skin [31,32,33] and reduction in the density and length of corneal nerve fibers [32,34]. Swelling of the dorsal root ganglion neurons and accumulation of Gb3 in the cytoplasm of these cells were also reported in human autopsy samples [35,36] and in α-galactosidase knock out animals [28,29]. In human sural nerve biopsy material ultrastructural morphometric analysis showed a reduction of the number of both small diameter myelinated axons and unmyelinated axons [36] and appearance of regenerating clusters was also reported [36,37]. Although numerous studies described alterations in the heat or cold thresholds of cutaneous sensory nerves [38,39] and the reduced mechanical sensitivity of the cornea [32,34], observations reporting the specific impairment of chemosensitive C-fiber neurons is sparse. One research group described a reduced flare reaction of the skin induced by subcutaneous administration of capsaicin in patients with Fabry disease [31,40].

The pathophysiology of the altered nociceptive functions observed in patients affected by the Fabry disease and in the corresponding animal models is not fully resolved [41]. Although it is plausible to assume that accumulation of the breakdown products of the glycosphingolipid catabolism may be an important causative factor, significant accumulation of lipids in the axons of peripheral nerves (especially in unmyelinated fibers) has been rarely observed. In biopsy specimens obtained from affected patients, accumulation of lipids in form of storage vesicles and lamellated bodies is prevalent in vascular endothelial cells and fibroblasts (perineural and endoneurial) [36,37]. Despite the less conspicuous accumulation of glycosphingolipids in the peripheral nociceptive axons the significance of the disturbed glycosphingolipid metabolism in nociceptive processing has been supported by some recent findings. Increased expression of the nociceptive ion channel transient receptor potential vanilloid type 1 (TRPV1) [42] and enhancement of voltage-gated calcium currents [43] have been demonstrated in experimental models of Fabry’s disease. Recent observations suggest a direct connection with the metabolic changes, accumulation of Gb3 and Gb4 and their metabolite, lyso-Gb3, and the increased activity of nociceptive neurons. Allodynia and reduced nociceptive thresholds following intraplantar injections of Gb3 and lyso-Gb3 have also been demonstrated [43]. In vitro experiments showed an increased activity of voltage-gated calcium channels in cultured primary sensory neurons following administration of these compounds [43]. Increased expression of TRPV1 in the dorsal root ganglion neurons and intraepidermal nerve endings have also been demonstrated in a genetic mouse model of this disease [42]. However, recent findings using also an α-galactosidase knockout mouse model did not confirm this observation by showing an unaltered expression of TRPV1 in dorsal root ganglion neurons, though they found a reduced thermal nociceptive behavioral response [29].

Taken together, these findings suggest that besides GM1, other glycosphingolipids such as Gb3 might also be involved in the modulation of the function of nociceptive ion channels in neuropathic pain. In addition, recent findings disclosed the role of a decreased activity of β-galactosidase, a key enzyme in ganglioside degradation, in inducing neuropathic pain in man [44].

Ganglioside species expressed by neurons of the peripheral nervous system may serve also as antigens in antibody and complement mediated immune reactions. Guillain-Barré syndrome is a relatively frequent neurological disease causing symmetrical, usually reversible paralysis and sensory disturbances. Neurally expressed gangliosides are involved in the pathogenesis of the so-called axonal types of Guillain-Barré syndrome, acute motor, or motor and sensory autoimmune neuropathy (AMAN and AMSAN) affecting predominantly the spinal nerves and the Miller-Fisher syndrome affecting predominantly cranial nerves [10,45]. Autoantigens produced by the mechanism of molecular mimicry bind to the axolemma at the nodes of Ranvier and paranodium where GM1 and GD1a gangliosides are concentrated [46]. Some human pathogenic bacteria such as Campylobacter jejuni express ganglioside-like lipooligosacharides which mimic the structure of human gangliosides [47] and trigger secretion of antibodies cross reactive with certain ganglioside species. The increased production of anti-ganglioside antibodies, mostly of IgM or IgG isotypes [48], initiate immune attack against axolemmal gangliosides of myelinated motor and sensory axons causing the activation of the complement cascade and macrophages. These events disrupt the connections between the axolemma and the myelin loops at the paranodal regions. Disruption of the axon-myelin contact sites results in dispersion of membrane proteins, including ion channels, and allows the invasion of macrophages into the periaxonal space resulting in slowing or blockade of action potential propagation and corresponding functional impairments of motor and sensory functions. Furthermore, local immune reaction and macrophage activity can induce axonal degeneration, too [10]. In case of Miller-Fisher syndrome the target antigen is the ganglioside GQ1b localized in the terminal segments of the motor axons [49]. Accordingly, this pathology affects the neuromuscular synapse and results in impairments of the neuromuscular transmission.

## 4. Effect of Peripheral Nerve Lesions on the Distribution of Ganglioside GM1 in Sensory Ganglia and Spinal Cord

Primary sensory neurons comprise morphologically, functionally and neurochemically differing populations of neurons subserving the transmission of information from sensory receptors to the central nervous system. In particular, primary sensory neurons which transmit nociceptive impulses are characterized by their exquisite sensitivity to capsaicin, the pungent principle in red peppers [50,51] and express the TRPV1 receptor [52]. TRPV1 is a molecular integrator of different nociceptor stimuli including noxious heat, acidic pH, and capsaicin. It is significantly involved in the mediation of heat hyperalgesia and inflammatory pain, too [53,54,55,56,57]. TRPV1 is the archetypal nociceptive ion channel and is expressed by practically all nociceptive primary sensory neurons which, in the rat, are mostly polymodal nociceptors [58,59]. This particular class of nociceptive primary sensory neurons can be divided into two separate groups: one population is made up of neurons containing sensory neuropeptides such as calcitonin gene-related peptide (CGRP) and substance P (SP). The other population is non-peptidergic and specifically binds the isolectin B4 from Bandeiraea simplicifolia [60,61,62]. Capsaicin-/chemosensitive peptidergic primary sensory neurons represent a unique population of nociceptive primary sensory neurons by possessing a dual function: on the one hand, they transmit nociceptive impulses towards the central nervous system (sensory afferent function), on the other hand, by the release of vasoactive neuropeptides, such as CGRP and SP from their peripheral nerve endings, they are involved in local regulation of tissue reactions, e.g., vasodilatation, plasma extravasation, smooth muscle contraction and immune function (local regulatory/sensory-efferent function) [51,55,63,64,65,66,67]. Importantly, the chemical phenotype(s) of primary sensory neurons cannot be regarded as a fixed property, but, on the contrary, a dynamic, ”on-demand” changing character of the neuron. Hence, injury inflicted upon the peripheral processes of primary sensory neurons may dramatically change their chemical phenotype, e.g., by changing their peptide expression pattern. This ”messenger plasticity” of primary sensory neurons has been amply demonstrated under different pathological conditions of peripheral nerve injury or tissue inflammation [68,69,70]. Messenger plasticity is a fundamental feature of nociceptive primary sensory neurons which are sensitive to capsaicin, the pungent agent in red peppers (paprika) [50] and express the archetypal nociceptive ion channel, the TRPV1 or capsaicin receptor [51]. Injuries inflicted upon a peripheral nerve resulting in neurotmesis [71], chemical axotomy by capsaicin [67,72,73,74,75] or causing neuropathic pain-like symptoms [76,77,78] are associated with profound changes in neuronal gene expression. The most notable changes (in the rat) comprise down-regulation of SP, CGRP, fluoride resistant acid phosphatase/thiamine monophosphatase [79,80,81,82,83], IB4 binding glycoprotein [84] and, importantly, TRPV1 [85,86]. In contrast, ”injury peptides” (cf. [68,69,82]) e.g., galanin [87], vasoactive intestinal polypeptide [88] and neuropeptide Y [89] are up-regulated after peripheral nerve injuries.

Injury of peripheral nerves is associated not only with chemical but also structural changes in the domain of primary sensory neurons. Studies conducted with classical silver impregnation techniques to demonstrate degenerating nerve fibers and endings disclosed moderate degeneration and loss of spinal afferent axons and axon terminals a few weeks after mechanical or chemical nerve injuries. [73,90,91,92,93]. Available experimental evidence indicate that this may be accounted for by a partial loss of dorsal root ganglion neurons relating to the affected nerve [73,90,94,95].

Hodological studies using CTB and its conjugates e.g., CTB-HRP suggested robust structural changes in the spinal dorsal horn after peripheral nerve injuries [96,97,98]. The most prominent feature of these changes involved markedly increased labeling of the substantia gelatinosa of the spinal dorsal horn by transganglionically transported CTB-HRP injected into an injured but not an intact sciatic nerve. This phenomenon was interpreted in terms of a sprouting response of injured myelinated A-fiber spinal afferents which normally do not project to the substantia gelatinosa [96,98,99,100]. This conclusion assumed that CTB is selectively bound and transported by myelinated nerve fibers [25]. However, it has been later revealed that injured but not intact unmyelinated nerve fibers do indeed bind CTB [101,102,103,104,105,106]. Electron microscopic histochemical studies on dorsal roots relating to injured but not intact sciatic nerves furnished direct evidence for the uptake and transport of CTB-HRP by unmyelinated dorsal root axons [104,107]. Hence, it has been concluded that following nerve injury, increased transganglionic labeling of the substantia gelatinosa by CTB and its conjugates can be accounted for by a switch of the chemical phenotype of C-fiber primary sensory neurons emitting unmyelinated C-fibers which project to the substantia gelatinosa, rather than a sprouting response of A-fiber afferents [101,102,103,104,106,107].

Although the specific binding of CTB to the GM1 ganglioside is well established [14,15], a possible role of this ganglioside species in sensory functions remained unclear. Electron microscopic histochemical studies demonstrated a conspicuous membrane localization of CTB-HRP [104,107,108] and suggested its association with membrane lipid rafts [104,107]. Inferred from the morphological observations, these findings indicated a putative role of GM1 ganglioside in the function of nociceptive primary sensory neurons possibly through interaction with membrane lipid microdomains/lipid rafts [104,107,108] (vide infra).

## 5. Ganglioside Species Involved in Pain Mechanisms

Although biochemical and histochemical studies demonstrated the localization of distinct ganglioside species in neuronal structures known to be involved in the transmission of nociceptive impulses, relatively few studies attempted to clarify the functional significance of gangliosides in nociceptive functions. Studies on the effects of morphine in cultured primary sensory neurons implicated ganglioside GM1 in the mechanisms of morphine’s excitatory action on these neurons. Supersensitivity of sensory neurons to morphine developing after chronic opioid exposure has been shown to be mediated by an increase in cellular GM1 level in primary sensory neurons and, in turn, CTB inhibited morphine’s excitatory action and also attenuated morphine tolerance [109,110,111].

Other studies implicated ganglioside GD2 in the mechanism of mechanical allodynia by showing decreased thresholds of withdrawal responses to mechanical but not thermal stimuli after intravenous or intrathecal administration of a GD2 antibody [112]. Single fiber recording revealed increased background activity of small sensory fibers and decreased mechanical threshold for Aδ afferents [113]. Prior intrathecal injection of capsaicin, which eliminates nociceptive C-fiber primary afferents [67] and decreases CGRP in the spinal dorsal horn, completely eliminated mechanical hyperalgesia [112].

Studies on the possible role of gangliosides in nociceptive mechanisms also used genetically engineered mice lacking enzymes involved in the synthesis of specific gangliosides. Mice with deletion of the St8sia1 gene lacking GD3 synthase and expressing only asialo- and a-series gangliosides displayed thermal hyperalgesia and mechanical allodynia and reduced nociceptive behavior during the late phase of the formalin test [114]. The decreased nociceptive thresholds for mechanical and thermal stimuli may be explained by a decrease of GD2 in GD3 synthase knock out mice, since peripheral administration of GD2 antibodies causes spontaneous pain in man [115] and mechanical allodynia in rats [112]. In addition, in cultured rat dorsal root ganglion neurons, GM1 has been shown to modulate opioid receptor signaling resulting in paradoxical hyperalgesia after administration of very low doses of opioid agonists [109]. In apparent contradiction to these findings demonstrating reduced nociceptive thresholds in in GD3 synthase knock out mice, reduced nocifensive behavior has been observed in the late phase of the formalin test. This might be explained through central actions of GM1 ganglioside. In GD3 synthase knock out mice, an increased expression of ganglioside GM1 has been detected which resulted in the attenuation of allodynia and hyperalgesia in a nerve constriction model of chronic pain [114,116]. An antinociceptive effect of intrathecally administered ganglioside GM1 has also been demonstrated in rats with chronic constriction injury of the sciatic nerve [116]. Moreover, systemically administered GM1 also resulted in an attenuation of peripheral nerve injury-induced hyperalgesia and abnormal nociceptive behavior [117]. It was hypothesized that GM1 attenuated increased neural activity by protecting spinal neurons from excitotoxic effects that may occur after peripheral nerve injury. Inhibition of the translocation of protein kinase C from the cytoplasm to the plasma membrane and attenuation of post-injury excitatory neurotoxicity by chelation of (increased) intracellular Ca^2+^ have also been implicated in the mechanism of action of GM1 [118].

Behavioral studies furnished evidence for a pain-producing effect of some gangliosides. Intraplantar injection of the b-series ganglioside GT1b produced nociceptive responses and enhanced formalin-induced nociception. GT1b-induced hyperalgesia was inhibited by N-methyl-d-aspartic acid receptor and type 1 metabotropic glutamate receptor antagonists, suggesting the involvement of glutamate receptors. Intraplantar injection of sialidase attenuated the late phase of formalin-induced nociception, further suggesting the involvement of endogenous gangliosides in nociceptive mechanisms [119].

Studies employing pharmacological manipulation of ganglioside synthesis provided further evidence for the involvement of gangliosides, in particular ganglioside GM1 in the regulation of function of nociceptive primary sensory neurons. Gangliosides are integral components of membrane lipid rafts which turned out to be important cellular entities of molecular assemblies involving nociceptive ion channels. Studies elucidating the role of membrane lipid rafts and their separate components, such as ganglioside GM1 and cholesterol implicated in nociceptive mechanisms will be described in the next section (vide infra).

## 6. Interaction of TRP Receptors with Membrane Lipid Rafts

Membrane lipid rafts are small (10–200 nm), heterogeneous, highly dynamic, sterol- and sphingolipid-enriched domains that compartmentalize cellular (membrane) processes [120,121,122]. Studies employing pharmacological manipulation of membrane cholesterol level have revealed that depletion of cholesterol by methyl-β-cyclodextrin markedly reduced TRPV1-mediated capsaicin- and proton-activated currents in cultured primary sensory neurons. Depletion of membrane cholesterol largely reduced membrane labeling of TRPV1 as assessed by immunohistochemistry and measurement of TRPV1 protein in membrane fractions [123]. It has been concluded that membrane cholesterol may determine the activity and amount of membrane TRPV1 [123] and ankyrin type 1 transient receptor potential TRPA1 channel [4] which may be localized in cholesterol-rich microdomains in nociceptive primary sensory neurons.

Gangliosides, including ganglioside GM1, are major integral components of membrane lipid rafts and participate in the targeted segregation of membrane proteins on the cell surface facilitating interactions and multiunit complex formation of specific membrane proteins, such as ion channels and receptors. Membrane ganglioside GM1 can be reliably and specifically visualized by the unique binding of CTB to this ganglioside species [14,15,16]. CTB and its conjugates with fluorescence dyes or HRP can be identified and visualized using fluorescence or enzyme histochemistry, respectively.

In cultured primary sensory neurons, inhibition by d-threo-1-phenyl-2-decanoylamino-3-morpholino-1-propanol (d-PDMP) of the rate limiting enzyme of ganglioside synthesis, GCS, permits the study of the role of gangliosides in neuronal processes [124]. Confocal microscopic observations of neuronal CTB binding of cultured adult rat primary sensory neurons in control and d-PDMP pretreated cultures showed a massive depletion of ganglioside GM1 from the neuronal membrane after d-PDMP [125]. Study of the capsaicin-induced activation of the TRPV1 receptor using the cobalt uptake method revealed a significant decrease in the proportion of activated neurons following d-PDMP pretreatment. Moreover, the proportion of TRPV1-immunoreactive neurons was also significantly reduced. These effects of GCS inhibition were reversible after cessation of the d-PDMP treatment. It has been concluded, therefore, that gangliosides, in particular GM1 may play a major role in the activation, and in the regulation of expression of the archetypal nociceptive ion channel, TRPV1 [125,126,127]. Capsaicin-induced release of CGRP was also significantly reduced after inhibition of GCS, indicating an impairment of the local regulatory, sensory-efferent function of primary sensory neurons [125]. These findings were interpreted in terms of an effect of inhibition of GCS on the composition of membrane lipid microdomains (rafts), i.e., depletion of gangliosides, in particular GM1. It has been suggested that an interaction of TRPV1 with membrane lipid raft components significantly participates in the activation of the TRPV1 receptor [125,126]. It has also been assumed that reduction of the expression of TRPV1 after inhibition of GCS may be attributed to an altered responsiveness of the peptidergic primary sensory neurons to nerve growth factor (NGF), which is critically involved in the regulation of TRPV1 expression [128,129,130]. Through an effect on TrkA, the high affinity receptor of NGF, ganglioside GM1 is implicated in the mechanism of action of NGF [131]. Depletion of membrane ganglioside GM1 inhibited NGF-induced autophosphorylation of TrkA and prevented the activation of downstream targets of TrkA-initiated intracellular protein kinase cascades [124]. Interestingly, in PC12 cells, genetically engineered overexpression of ganglioside GM1 resulted in a massive reduction of the NGF responsiveness and inhibition of neurite outgrowth. These changes were associated with a marked reduction in NGF-induced TrkA dimerization and phosphorylation, a crucial process in the activation of downstream signaling pathways of NGF. It has been suggested that the effects of ganglioside GM1 are concentration dependent, higher intracellular concentrations being inhibitory through an action on the properties of the membrane lipid rafts and the intracellular localization of NGF receptors and relevant signaling molecules [132].

Recent studies confirmed and extended the original observations concerning the role of ganglioside GM1 in the mechanism of activation of nociceptive TRP receptors. An inhibitory effect on the activation of TRPV1 following the selective inhibition of GCS by d-PDMP was also observed in cultured trigeminal ganglion neurons and in Chinese hamster ovary (CHO) cell lines stably expressing the rat TRPV1 channel [133]. These data indicate that reduction of glycosphingolipid synthesis attenuates nociceptor activation not only of spinal, but also of trigeminal nociceptive primary sensory neurons. It must be mentioned, however, that in this study cell cultures were incubated for a relatively short period of time (“overnight”) with d-PDMP. Since the magnitude of the depletion of glycosphingolipids, most importantly GM1, was not determined, it is plausible to assume, based on previous data on the time course of d-PDMP-induced GM1 depletion [125] that this treatment did not produce a substantial depletion of gangliosides. Nevertheless, the authors reported highly significant reductions in both capsaicin- and resiniferatoxin-induced Ca^2+^ uptake in the treated cells.

The effects of myriocin, a selective inhibitor of serine-palmitoyl transferase, the rate limiting enzyme of sphingolipid synthesis [134] was also investigated on the activation of different nociceptive TRP channels [133,135]. Though it has been claimed that this treatment depletes gangliosides from the plasma membrane (“ganglioside biosynthesis inhibitor”; “ganglioside breakdown by myriocin”) the conclusions based on this assumption need to be treated with caution because of the simultaneous effects (reduction) by myriocin of non-glycoconjugated derivates of sphingosine, such as ceramide and sphingomyelin. These latter lipids are also major constituents not only of membrane lipid rafts but even of the postulated third type of membrane microdomains, the ceramide-rich platforms, CRPs [121]. Hence, the inhibition of the biosynthesis of non-glycoconjugated derivates of sphingosine *per se* could affect the integrity of lipid rafts, independently of the action of myriocin on glycosphingolipids, including gangliosides. Indeed, several studies demonstrated that treatment of sensory ganglion cell cultures or TRPV1 expressing cells with sphingomyelinase, which cleaves sphingomyelin but not glycoconjugated sphingolipids, profoundly reduced TRPV1 (and TRPA1) activation [133,135]. Data obtained using sphingomyelinase treatment suggest, that besides cholesterol and gangliosides, the sphingomyelin level in the membrane rafts is also critical in maintaining the integrity and functions of raft-embedded molecular complexes including the operation of TRP channels [133,135]. It cannot be excluded, however, that manipulation of the sphingomyelin level, and consequently, ceramide concentration in the plasma membrane after sphingomyelinase treatment could indirectly influence the concentration (and distribution) of glycosphingolipids as well, but detailed description on the dynamics of changes in the membrane lipid composition remains to be clarified. Importantly, these studies disclosed that apart from activation of TRPV1, the activation of other nociceptive transducer molecules, such as TRPA1 and the effects of endogenous/exogenous algogenic substances which activate them could be affected by disintegration of membrane lipid rafts and/or manipulation of the metabolism of raft components, including probably gangliosides as well [135]. The findings that the TRPA1 agonist, allyl isothiocyanate-induced cobalt uptake was significantly reduced after chronic d-PDMP treatment demonstrate that activation of this nociceptive ion channel is also dependent on membrane gangliosides [136].

The association of TRPV1 with the specific membrane protein caveolin-1, which plays a pivotal role in endocytotic processes has also been demonstrated. Experiments on a CHO cell-based expression system demonstrated restricted mobility and association of TRPV1 with caveolin-1 [137]. Importantly, exposure of TRPV1-expressing cells to vanilloid receptor agonist resiniferatoxin resulted in the translocation of TRPV1 into cytoplasmic caveolar vesicles. This observation strongly suggested that association of TRPV1 with caveolin-1 and the caveolin-1-dependent internalization of TRPV1 may be a possible mechanism of vanilloid agonist-induced desensitization of the TRPV1 receptor [137]. It is worth noting that NGF-, insulin-like growth factor-1 (IGF-1)- and insulin-induced sensitization of the TRPV1 receptor to capsaicin [138] has also been shown to be mediated by translocation and insertion of intracellular TRPV1 into the plasma membrane [139,140]. Both NGF and insulin/IGF signaling is critically dependent on the functions of lipid rafts [131,141], and in the case of insulin on the caveolar membrane [142,143]. The substantial role of the stimulus intensity-dependent internalization and dynamic recycling of membrane-bound TRPV1 has been recently confirmed and supplemented with further details demonstrating the importance of synaptotagmin 1 and 7 in the mechanism of capsaicin-induced tachyphylaxis and recovery [144].

## 7. Conclusion and Perspectives

Experimental data on the role of gangliosides in somatosensation are relatively sparse as compared to the vast literature on the effects of these glycosphingolipids on the central nervous system. However, investigations into the mechanisms of somatosensory functions, in particular transmission of nociceptive impulses have revealed that glycosphingolipids, including gangliosides may interfere with the function of primary sensory neurons through diverse interactions with membrane receptors/ion channels, lipid rafts, membrane, and intracellular signaling pathways, cellular calcium homeostasis, and immune mechanisms. Ganglioside GM1 plays an important role in the NGF-dependent regulation of the expression and activation of nociceptive ion channels, such as the archetypal TRPV1 receptor. Major ganglioside species GM1, GD1a, GD1b, and GT1b are involved in the modulation of spinal and trigeminal nociception either by contributing to the functional organization of membrane lipid rafts or linking membrane proteins to membrane and intracellular signaling pathways.

Importantly, alterations in cellular ganglioside homeostasis may lead to pathological changes, such as peripheral neuropathies affecting the pain system. Persistent increase in neuronal ganglioside GM1 level has been demonstrated after peripheral nerve lesions as well as after perineural treatment with vanilloid compounds capsaicin and resiniferatoxin resulting in prolonged thermo- and chemoanalgesia. The lesion-induced elevation of neuronal GM1 level under these conditions resembles ganglioside storage disorders. Further studies are warranted to clarify whether increased level of GM1 ganglioside in nociceptive primary sensory neurons under these conditions may be accounted for by an increased synthesis or decreased degradation of the ganglioside. Further studies using mice with targeted conditional knock out of selected genes involved in the synthesis or degradation of specific gangliosides expressed in nociceptive primary sensory neurons may provide further support for the role of gangliosides in peripheral pain mechanisms. Pharmacological manipulation of neuronal ganglioside level for potential therapeutic purposes remains an intriguing possibility to be investigated for future research.

## Abbreviations

CGRP	Calcitonin gene-related peptide
CHO	Chinese hamster ovary
CRP	Ceramide-rich platform
CTB	Choleratoxin B subunit
D-PDMP	D-threo-1-phenyl-2-decanoylamino-3- morpholino-1-propanol
HRP	Horse radish peroxidase
IGF-1	Insulin-like growth factor
NGF	Nerve growth factor
SP	Substance P
TRP	Transient receptor potential
TRPA1	Transient receptor potential ankyrin-1
TRPV1	Transient receptor potential vanilloid type 1

## References

[1] Sezgin E., Levental I., Mayor S., Eggeling C. (2017). The mystery of membrane organization: Composition, regulation and roles of lipid rafts. Nat. Rev. Mol. Cell Biol..

[2] Tsui-Pierchala B.A., Encinas M., Milbrandt J., Johnson M. (2002). Lipid rafts in neuronal signaling and function. Trends Neurosci..

[3] Taberner F.J., Fernández-Ballester G., Fernández-Carvajal A., Ferrer-Montiel A. (2015). TRP channels interaction with lipids and its implications in disease. Biochim. Biophys. Acta Biomembr..

[4] Startek J.B., Boonen B., López-Requena A., Talavera A., Alpizar Y.A., Ghosh D., van Ranst N., Nilius B., Voets T., Talavera K. (2019). Mouse TRPA1 function and membrane localization are modulated by direct interactions with cholesterol. Elife.

[5] Paratcha G., Ibánez C.F. (2002). Lipid rafts and the control of neurotrophic factor signaling in the nervous system: Variations on a theme. Curr. Opin. Neurobiol..

[6] Kappagantula S., Andrews M.R., Cheah M., Abad-Rodriguez J., Dotti C.G., Fawcett J.W. (2014). Neu3 sialidase-mediated ganglioside conversion is necessary for axon regeneration and is blocked in CNS axons. J. Neurosci..

[7] Lopez P.H.H., Báez B.B. (2018). Gangliosides in Axon Stability and Regeneration. Progress in Molecular Biology and Translational Science.

[8] Sandhoff R., Schulze H., Sandhoff K. (2018). Ganglioside Metabolism in Health and Disease. Progress in Molecular Biology and Translational Science.

[9] Yu R.K., Tsai Y.T., Ariga T., Yanagisawa M. (2011). Structures, biosynthesis, and functions of gangliosides-an overview. J. Oleo Sci..

[10] Schnaar R.L., Gerardy-Schahn R., Hildebrandt H. (2014). Sialic acids in the brain: Gangliosides and polysialic acid in nervous system development, stability, disease, and regeneration. Physiol. Rev..

[11] Svennerholm L. (1964). The gangliosides. J. Lipid Res..

[12] Yamashita T., Wada R., Sasaki T., Deng C., Bierfreund U., Sandhoff K., Proia R.L. (1999). A vital role for glycosphingolipid synthesis during development and differentiation. Proc. Natl. Acad. Sci. USA.

[13] Jennemann R., Sandhoff R., Wang S., Kiss E., Gretz N., Zuliani C., Martin-Villalba A., Jäger R., Schorle H., Kenzelmann M. (2005). Cell-specific deletion of glucosylceramide synthase in brain leads to severe neural defects after birth. Proc. Natl. Acad. Sci. USA.

[14] Cuatrecasas P. (1973). Gangliosides and membrane receptors for cholera toxin. Biochemistry.

[15] Holmgren J., Lonnroth I., Svennerholm L. (1973). Fixation and inactivation of cholera toxin by GM1 ganglioside. Scand. J. Infect. Dis..

[16] King C.A., Heyningen W.E.V. (1973). Deactivation of cholera toxin by a sialidase-resistant monosialosylganglioside. J. Infect. Dis..

[17] Robertson B., Grant G. (1989). Immunocytochemical evidence for the localization of the GM1 ganglioside in carbonic anhydrase-containing and RT 97-immunoreactive rat primary sensory neurons. J. Neurocytol..

[18] Todd A.J. (2010). Neuronal circuitry for pain processing in the dorsal horn. Nat. Rev. Neurosci..

[19] Kotani M., Kawashima I., Ozawa H., Terashima T., Tai T. (1993). Differential distribution of major gangliosides in rat central nervous system detected by specific monoclonal antibodies. Glycobiology.

[20] Gong Y., Tagawa Y., Lunn M.P., Laroy W., Heffer-Lauc M., Li C.Y., Griffin J.W., Schnaar R.L., Sheikh K. (2002). Localization of major gangliosides in the PNS: Implications for immune neuropathies. Brain.

[21] Vajn K., Viljetić B., Degmečić I.V., Schnaar R.L., Heffer M. (2013). Differential Distribution of Major Brain Gangliosides in the Adult Mouse Central Nervous System. PLoS ONE.

[22] Jancsó G., Király E. (1980). Distribution of chemosensitive primary sensory afferents in the central nervous system of the rat. J. Comp. Neurol..

[23] Szentágothai J. (1964). Neuronal and synaptic arrangement in the substantia gelatinosa rolandi. J. Comp. Neurol..

[24] Harper A.A., Lawson S.N. (1985). Electrical properties of rat dorsal root ganglion neurones with different peripheral nerve conduction velocities. J. Physiol..

[25] Robertson B., Grant G. (1985). A comparison between wheat germ agglutinin-and choleragenoid-horseradish peroxidase as anterogradely transported markers in central branches of primary sensory neurones in the rat with some observations in the cat. Neuroscience.

[26] Politei J.M., Bouhassira D., Germain D.P., Goizet C., Guerrero-Sola A., Hilz M.J., Hutton E.J., Karaa A., Liguori R., Üçeyler N. (2016). Pain in Fabry Disease: Practical Recommendations for Diagnosis and Treatment. CNS Neurosci. Ther..

[27] Üçeyler N., Biko L., Hose D., Hofmann L., Sommer C. (2016). Comprehensive and differential long-term characterization of the alpha-galactosidase A deficient mouse model of Fabry disease focusing on the sensory system and pain development. Mol. Pain.

[28] Hofmann L., Hose D., Grießhammer A., Blum R., Döring F., Dib-Hajj S., Waxman S., Sommer C., Wischmeyer E., Üçeyler N. (2018). Characterization of small fiber pathology in a mouse model of fabry disease. Elife.

[29] Namer B., Ørstavik K., Schmidt R., Mair N., Kleggetveit I.P., Zeidler M., Martha T., Jorum E., Schmelz M., Kalpachidou T. (2017). Changes in ionic conductance signature of nociceptive neurons underlying Fabry disease phenotype. Front. Neurol..

[30] Ohshima T., Murray G.J., Swaim W.D., Longenecker G., Quirk J.M., Cardarelli C.O., Sugimoto Y., Pastan I., Gottesman M.M., Brady R.O. (1997). α-Galactosidase A deficient mice: A model of Fabry disease. Proc. Natl. Acad. Sci. USA.

[31] Torvin Møller A., Winther Bach F., Feldt-Rasmussen U., Rasmussen Å., Hasholt L., Lan H., Sommer C., Kølvraa S., Ballegaard M., Staehelin Jensen T. (2009). Functional and structural nerve fiber findings in heterozygote patients with Fabry disease. Pain.

[32] Tavakoli M., Marshall A., Thompson L., Kenny M., Waldek S., Efron N., Malik R.A. (2009). Corneal confocal microscopy: A novel noninvasive means to diagnose neuropathy in patients with Fabry disease. Muscle Nerve.

[33] Üçeyler N., Kahn A.-K., Kramer D., Zeller D., Casanova-Molla J., Wanner C., Weidemann F., Katsarava Z., Sommer C. (2013). Impaired small fiber conduction in patients with Fabry disease: A neurophysiological case–control study. BMC Neurol..

[34] Bitirgen G., Turkmen K., Malik R.A., Ozkagnici A., Zengin N. (2018). Corneal confocal microscopy detects corneal nerve damage and increased dendritic cells in Fabry disease. Sci. Rep..

[35] Kahn P. (1973). Anderson Fabry disease: A histopathological study of three cases with observations on the mechanism of production of pain. J. Neurol. Neurosurg. Psychiatry.

[36] Toyooka K., Said G. (1997). Nerve biopsy findings in hemizygous and heterozygous patients with Fabry’s disease. J. Neurol..

[37] Gayathri N., Yasha T., Kanjalkar M., Agarwal S., Chandrashekar Sagar B., Santosh V., Shankar S. (2008). Fabry’s disease: An ultrastructural study of nerve biopsy. Ann. Indian Acad. Neurol..

[38] Dütsch M., Marthol H., Stemper B., Brys M., Haendl T., Hilz M.J. (2002). Small fiber dysfunction predominates in Fabry neuropathy. J. Clin. Neurophysiol..

[39] Luciano C.A., Russell J.W., Banerjee T.K., Quirk J.M., Scott L.J.C., Dambrosia J.M., Barton N.W., Schiffmann R. (2002). Physiological characterization of neuropathy in Fabry’s disease. Muscle Nerve.

[40] Moller A.T., Feldt-Rasmussen U., Rasmussen A.K., Sommer C., Hasholt L., Bach F.W., Kolvraa S., Jensen T.S. (2006). Small-fibre neuropathy in female Fabry patients: Reduced allodynia and skin blood flow after topical capsaicin. J. Peripher. Nerv. Syst..

[41] Biegstraaten M., Hollak C.E.M., Bakkers M., Faber C.G., Aerts J.M.F.G., van Schaik I.N. (2012). Small fiber neuropathy in Fabry disease. Mol. Genet. Metab..

[42] Lakomá J., Rimondini R., Montiel A.F., Donadio V., Liguori R., Caprini M. (2016). Increased expression of trpv1 in peripheral terminals mediates thermal nociception in Fabry disease mouse model. Mol. Pain.

[43] Choi L., Vernon J., Kopach O., Minett M.S., Mills K., Clayton P.T., Meert T., Wood J.N. (2015). The Fabry disease-associated lipid lyso-Gb3 enhances voltage-gated calcium currents in sensory neurons and causes pain. Neurosci. Lett..

[44] Darin N., Kyllerman M., Hård A.L., Nordborg C., Månsson J.E. (2009). Juvenile galactosialidosis with attacks of neuropathic pain and absence of sialyloligosacchariduria. Eur. J. Paediatr. Neurol..

[45] Hughes R.A.C., Cornblath D.R. (2005). Guillain-Barré syndrome. Lancet.

[46] Corbo M., Quattrini A., Latov N., Hays A.P. (1993). Localization of GM1 and gal(β1-3)galnac antigenic determinants in peripheral nerve. Neurology.

[47] Yuki N., Odaka M. (2005). Ganglioside mimicry as a cause of Guillain-Barré syndrome. Curr. Opin. Neurol..

[48] Deisenhammer F., Keir G., Pfausler B., Thompson E.J. (1996). Affinity of anti-GM1 antibodies in Guillain-Barré syndrome patients. J. Neuroimmunol..

[49] Plomp J.J., Molenaar P.C., O’Hanlon G.M., Jacobs B.C., Veitch J., Daha M.R., Van Doorn P.A., Van Der Mecha F.G.A., Vincent A., Morgan B.P. (1999). Miller Fisher anti-GQ1b antibodies: α-Latrotoxin-like effects on motor end plates. Ann. Neurol..

[50] Jancsó G., Király E., Jancsó-Gábor A. (1977). Pharmacologically induced selective degeneration of chemosensitive primary sensory neurones. Nature.

[51] Jancsó G., Sántha P. (2015). The foundation of sensory pharmacology: Nicholas (Miklos) Jancso and the Szeged contribution. Temperature.

[52] Caterina M.J., Schumacher M.A., Tominaga M., Rosen T.A., Levine J.D., Julius D. (1997). The capsaicin receptor: A heat-activated ion channel in the pain pathway. Nature.

[53] Caterina M.J., Park U. (2006). Chapter 4 TRPV1: A Polymodal Sensor in the Nociceptor Terminal. Curr. Top. Membr..

[54] Caterina M.J., Julius D. (2001). The vanilloid receptor: A molecular gateway to the pain pathway. Annu. Rev. Neurosci..

[55] Nilius B., Szallasi A. (2014). Transient receptor potential channels as drug targets: From the science of basic research to the art of medicine. Pharmacol. Rev..

[56] Nagy I., Sántha P., Jancsó G., Urbán L. (2004). The role of the vanilloid (capsaicin) receptor (TRPV1) in physiology and pathology. Eur. J. Pharmacol..

[57] Davis J.B., Gray J., Gunthorpe M.J., Hatcher J.P., Davey P.T., Overend P., Harries M.H., Latcham J., Clapham C., Atkinson K. (2000). Vanilloid receptor-1 is essential for inflammatory thermal hyperalgesia. Nature..

[58] Lynn B. (1984). Effect of neonatal treatment with capsaicin on the numbers and properties of cutaneous afferent units from the hairy skin of the rat. Brain Res..

[59] Szolcsányi J., Anton F., Reeh P.W., Handwerker H.O. (1988). Selective excitation by capsaicin of mechano-heat sensitive nociceptors in rat skin. Brain Res..

[60] Price T.J., Flores C.M. (2007). Critical Evaluation of the Colocalization Between Calcitonin Gene-Related Peptide, Substance P, Transient Receptor Potential Vanilloid Subfamily Type 1 Immunoreactivities, and Isolectin B4 Binding in Primary Afferent Neurons of the Rat and Mouse. J. Pain.

[61] Snider W.D., McMahon S.B. (1998). Tackling pain at the source: New ideas about nociceptors. Neuron.

[62] Silverman J.D., Kruger L. (1988). Lectin and neuropeptide labeling of separate populations of dorsal root ganglion neurons and associated “nociceptor” thin axons in rat testis and cornea whole-mount preparations. Somatosens. Res..

[63] Holzer P. (1998). Implications of tachykinins and calcitonin gene-related peptide in inflammatory bowel disease. Digestion.

[64] Jancsó G., Chahl J., Szolcsányi J., Lembeck F. (1984). Sensory nerves as modulators of inflammatory reactions. Antidromic Vasodilatation and Neurogenic Inflammation.

[65] Maggi C.A., Meli A. (1988). The sensory-efferent function of capsaicin-sensitive sensory neurons. Gen. Pharmacol..

[66] Jancsó G., Király E., Such G., Joó F., Nagy A. (1987). Neurotoxic effect of capsaicin in mammals. Acta Physiol. Hung..

[67] Jancsó G., Oszlács O., Sántha P. (2012). The Capsaicin Paradox: Pain Relief by an Algesic Agent. Antiinflamm. Antiallergy. Agents Med. Chem..

[68] Hökfelt T., Zhang X., Wiesenfeld-Hallin Z. (1994). Messenger plasticity in primary sensory neurons following axotomy and its functional implications. Trends Neurosci..

[69] Nahin R.L., Ren K., De León M., Ruda M. (1994). Primary sensory neurons exhibit altered gene expression in a rat model of neuropathic pain. Pain.

[70] Costigan M., Befort K., Karchewski L., Griffin R.S., D’Urso D., Allchorne A., Sitarski J., Mannion J.W., Pratt R.E., Woolf C.J. (2002). Replicate high-density rat genome oligonucleotide microarrays reveal hundreds of regulated genes in the dorsal root ganglion after peripheral nerve injury. BMC Neurosci..

[71] Sunderland S. (1951). A classification of peripheral nerve injuries producing loss of function. Brain.

[72] Gamse R., Holzer P., Lembeck F. (1980). Decrease of substance P in primary afferent neurones and impairment of neurogenic plasma extravasation by capsaicin. Br. J. Pharmacol..

[73] Jancsó G., Lawson S.N. (1990). Transganglionic Degeneration of Capsaicin-Sensitive C-Fiber Primary Afferent Terminals. Neuroscience.

[74] Gibson S.J., McGregor G., Bloom S.R., Polak J.M., Wall P.D. (1982). Local application of capsaicin to one sciatic nerve of the adult rat induces a marked depletion in the peptide content of the lumbar dorsal horn. Neuroscience.

[75] Jancsó G., Király E., Jancsó-Gábor A. (1980). Direct evidence for an axonal site of action of capsaicin. Naunyn. Schmiedebergs. Arch. Pharmacol..

[76] Bennett G.J., Chung J.M., Honore M., Seltzer Z. (2003). Models of neuropathic pain in the rat. Curr. Protoc. Pharmacol..

[77] Seltzer Z., Dubner R., Shir Y. (1990). A novel behavioral model of neuropathic pain disorders produced in rats by partial sciatic nerve injury. Pain.

[78] Kim S.H., Chung J.M. (1992). An experimental model for peripheral neuropathy produced by segmental spinal nerve ligation in the rat. Pain.

[79] Castro-Lopes J.M., Coimbra A., Grant G., Arvidsson J. (1990). Ultrastructural changes of the central scalloped (C1) primary afferent endings of synaptic glomeruli in the substantia gelatinosa Rolandi of the rat after peripheral neurotomy. J. Neurocytol..

[80] Gamse R., Petsche U., Lembeck F., Jancsó G. (1982). Capsaicin Applied to Peripheral-Nerve Inhibits Axoplasmic-Transport of Substance-P and Somatostatin. Brain Res..

[81] Knyihár E., Csillik B. (1976). Effect of peripheral anatomy on the fine structure and histochemistry of the Rolando substance: Degenerative atrophy of central processes of pseudounipolar cells. Exp. Brain Res..

[82] Jancsó G. (1992). Pathobiological reactions of C-fibre primary sensory neurones to peripheral nerve injury. Exp. Physiol..

[83] Colmant H.J. (1959). Aktivitätsschwankungen der sauren Phosphatase im Rückenmark und den Spinalganglien der Ratte nach Durchschneidung des Nervus ischiadicus. Arch. Psychiatr. Nervenkrankheiten.

[84] Hammond D.L., Ackerman L., Holdsworth R., Elzey B. (2004). Effects of spinal nerve ligation on immunohistochemically identified neurons in the L4 and L5 dorsal root ganglia of the rat. J. Comp. Neurol..

[85] Michael G.J., Priestley J.V. (1999). Differential expression of the mRNA for the vanilloid receptor subtype 1 in cells of the adult rat dorsal root and nodose ganglia and its downregulation by axotomy. J. Neurosci..

[86] Szigeti C., Sántha P., Körtvély E., Nyári T., Horváth V.J., Deák E., Dux M., Gulya K., Jancsó G. (2012). Disparate changes in the expression of transient receptor potential vanilloid type 1 receptor mRNA and protein in dorsal root ganglion neurons following local capsaicin treatment of the sciatic nerve in the rat. Neuroscience.

[87] Zhang X., Xu Z.Q., Shi T.J., Landry M., Holmberg K., Ju G., Tong Y.G., Bao L., Cheng X.P., Wiesenfeld-Hallin Z. (1998). Regulation of expression of galanin and galanin receptors in dorsal root ganglia and spinal cord after axotomy and inflammation. Proceedings of the Annals of the New York Academy of Sciences.

[88] Doughty S.E., Atkinson M.E., Shehab S.A.S. (1991). A quantitative study of neuropeptide immunoreactive cell bodies of primary afferent sensory neurons following rat sciatic nerve peripheral axotomy. Regul. Pept..

[89] Zhang X., Wiesenfeld-Hallin Z., Hökfelt T. (1994). Effect of Peripheral Axotomy on Expression of Neuropeptide Y Receptor mRNA in Rat Lumbar Dorsal Root Ganglia. Eur. J. Neurosci..

[90] Aldskogius H., Arvidsson J., Grant G. (1985). The reaction of primary sensory neurons to peripheral nerve injury with particular emphasis on transganglionic changes. Brain Res..

[91] Jancsó G., Ambrus A., Besson J.M., Guilbaud G., Ollat H. (1994). Capsaicin sensitivity of primary sensory neurones and its regulation. Peripheral neurons in nociception: Physio-pharmacological aspects.

[92] Grant G., Arvidsson J. (1975). Transganglionic degeneration in trigeminal primary sensory neurons. Brain Res..

[93] Knyihár-Csillik E., Rakic P., Csillik B. (1987). Transganglionic degenerative atrophy in the substantia gelatinosa of the spinal cord after peripheral nerve transection in rhesus monkeys. Cell Tissue Res..

[94] Himes B.T., Tessler A. (1989). Death of some dorsal root ganglion neurons and plasticity of others following sciatic nerve section in adult and neonatal rats. J. Comp. Neurol..

[95] Arvidsson J., Ygge J., Grant G. (1986). Cell loss in lumbar dorsal root ganglia and transganglionic degeneration after sciatic nerve resection in the rat. Brain Res..

[96] Woolf C.J., Shortland P., Reynolds M., Ridings J., Doubell T., Coggeshall R.E. (1995). Reorganization of central terminals of myelinated primary afferents in the rat dorsal horn following peripheral axotomy. J. Comp. Neurol..

[97] Mannion R.J., Doubell T.P., Gill H., Woolf C.J. (1998). Deafferentation is insufficient to induce sprouting of A-fibre central terminals in the rat dorsal horn. J. Comp. Neurol..

[98] Woolf C.J., Shortland P., Coggeshall R.E. (1992). Peripheral nerve injury triggers central sprouting of myelinated afferents. Nature.

[99] Lekan H.A., Carlton S.M., Coggeshall R.E. (1996). Sprouting of Aβ fibers into lamina II of the rat dorsal horn in peripheral neuropathy. Neurosci. Lett..

[100] Nakamura S.I., Myers R.R. (1999). Myelinated afferents sprout into lamina II of L3-5 dorsal horn following chronic constriction nerve injury in rats. Brain Res..

[101] Bao L., Wang H.F., Cai H.J., Tong Y.G., Jin S.X., Lu Y.J., Grant G., Hökfelt T., Zhang X. (2002). Peripheral axotomy induces only very limited sprouting of coarse myelinated afferents into inner lamina II of rat spinal cord. Eur. J. Neurosci..

[102] Shehab S.A., Spike R.C., Todd A.J. (2003). Evidence against cholera toxin B subunit as a reliable tracer for sprouting of primary afferents following peripheral nerve injury. Brain Res..

[103] Tong Y.G., Wang H.F., Ju G., Grant G., Hökfelt T., Zhang X. (1999). Increased uptake and transport of cholera toxin B-subunit in dorsal root ganglion neurons after peripheral axotomy: Possible implications for sensory sprouting. J. Comp. Neurol..

[104] Sántha P., Jancsó G. (2003). Transganglionic transport of choleragenoid by capsaicin-sensitive C-fibre afferents to the substantia gelatinosa of the spinal dorsal horn after peripheral nerve section. Neuroscience.

[105] Jancsó G., Sántha P., Gecse K. (2002). Peripheral nerve lesion-induced uptake and transport of choleragenoid by capsaicin-sensitive C-fibre spinal ganglion neurons. Acta Biol. Hung..

[106] Hughes D.I., Scott D.T., Todd A.J., Riddell J.S. (2003). Lack of evidence for sprouting of Abeta afferents into the superficial laminas of the spinal cord dorsal horn after nerve section. J. Neurosci..

[107] Jancsó G., Sántha P., Brune K., Handwerker H.O. (2004). Transganglionic transport of choleragenoid by injured C fibres to the substantia gelatinosa: Relevance to neuropathic pain and hyperalgesia. Hyperalgesia: Molecular Mechanisms and Clinical Implications.

[108] Oszlács O., Jancsó G., Kis G., Dux M., Sántha P. (2015). Perineural capsaicin induces the uptake and transganglionic transport of choleratoxin b subunit by nociceptive c-fiber primary afferent neurons. Neuroscience.

[109] Crain S.M., Shen K.F. (1998). GM1 ganglioside-induced modulation of opioid receptor-mediated functions. Ann. N.Y. Acad. Sci..

[110] Shen K.F., Crain S.M. (1992). Chronic selective activation of excitatory opioid receptor functions in sensory neurons results in opioid “dependence” without tolerance. Brain Res..

[111] Shen K.F., Crain S.M. (2001). Cholera toxin-B subunit blocks excitatory opioid receptor-mediated hyperalgesic effects in mice, thereby unmasking potent opioid analgesia and attenuating opioid tolerance/dependence. Brain Res..

[112] Sorkin L.S., Yu A.L., Junger H., Doom C.M. (2002). Antibody directed against GD(2) produces mechanical allodynia, but not thermal hyperalgesia when administered systemically or intrathecally despite its dependence on capsaicin sensitive afferents. Brain Res..

[113] Xiao W.H., Yu A.L., Sorkin L.S. (1997). Electrophysiological characteristics of primary afferent fibers after systemic administration of anti-GD2 ganglioside antibody. Pain.

[114] Handa Y., Ozaki N., Honda T., Furukawa K., Tomita Y., Inoue M., Okada M., Sugiura Y. (2005). GD3 synthase gene knockout mice exhibit thermal hyperalgesia and mechanical allodynia but decreased response to formalin-induced prolonged noxious stimulation. Pain.

[115] Yu A.L., Uttenreuther-Fischer M.M., Huang C.S., Tsui C.C., Gillies S.D., Reisfeld R.A., Kung F.H. (1998). Phase I trial of a human-mouse chimeric anti-disialoganglioside monoclonal antibody ch14.18 in patients with refractory neuroblastoma and osteosarcoma. J. Clin. Oncol..

[116] Mao J., Price D.D., Hayes R.L., Lu J., Mayer D.J. (1992). Intrathecal GM1 ganglioside and local nerve anesthesia reduce nociceptive behaviors in rats with experimental peripheral mononeuropathy. Brain Res..

[117] Mao J., Hayes R.L., Price D.D., Coghill R.C., Lu J., Mayer D.J. (1992). Post-injury treatment with GM1 ganglioside reduces nociceptive behaviors and spinal cord metabolic activity in rats with experimental peripheral mononeuropathy. Brain Res..

[118] Mao J., Price D.D., Mayer D.J., Hayes R.L. (1992). Pain-related increases in spinal cord membrane-bound protein kinase C following peripheral nerve injury. Brain Res..

[119] Watanabe S., Tan-No K., Tadano T., Higashi H. (2011). Intraplantar injection of gangliosides produces nociceptive behavior and hyperalgesia via a glutamate signaling mechanism. Pain.

[120] Pike L.J. (2006). Rafts defined: A report on the Keystone symposium on lipid rafts and cell function. J. Lipid Res..

[121] Bieberich E. (2018). Sphingolipids and lipid rafts: Novel concepts and methods of analysis. Chem. Phys. Lipids.

[122] Simons K., Ikonen E. (1997). Functional rafts in cell membranes. Nature.

[123] Liu M., Huang W., Wu D., Priestley J. (2006). V TRPV1, but not P2X, requires cholesterol for its function and membrane expression in rat nociceptors. Eur. J. Neurosci..

[124] Mutoh T., Tokuda A., Inokuchi J., Kuriyama M. (1998). Glucosylceramide synthase inhibitor inhibits the action of nerve growth factor in PC12 cells. J. Biol. Chem..

[125] Sántha P., Oszlács O., Dux M., Dobos I., Jancsó G. (2010). Inhibition of glucosylceramide synthase reversibly decreases the capsaicin-induced activation and TRPV1 expression of cultured dorsal root ganglion neurons. Pain.

[126] Jancsó G., Dux M., Oszlács O., Sántha P. (2008). Activation of the transient receptor potential vanilloid-1 (TRPV1) channel opens the gate for pain relief. Br. J. Pharmacol..

[127] Jancsó G., Oszlács O., Dobos I., Sántha P. (2008). Glucosylceramide synthase regulates the capsaicin sensitivity of cultured dorsal root ganglion neurons. 6th Forum Eur. Neurosci. Geneva Switz..

[128] Winter J., Forbes C.A., Sternberg J., Lindsay R.M. (1988). Nerve growth factor (NGF) regulates adult rat cultured dorsal root ganglion neuron responses to the excitotoxin capsaicin. Neuron.

[129] Bevan S., Winter J. (1995). Nerve growth factor (NGF) differentially regulates the chemosensitivity of adult rat cultured sensory neurons. J. Neurosci..

[130] Aguayo L.G., White G. (1992). Effects of nerve growth factor on TTX- and capsaicin-sensitivity in adult rat sensory neurons. Brain Res..

[131] Farooqui T., Franklin T., Pearl D.K., Yates A.J. (1997). Ganglioside GM1 enhances induction by nerve growth factor of a putative dimer of TrkA. J. Neurochem..

[132] Nishio M., Fukumoto S., Furukawa K., Ichimura A., Miyazaki H., Kusunoki S., Urano T., Furukawa K. (2004). Overexpressed GM1 suppresses nerve growth factor (NGF) signals by modulating the intracellular localization of NGF receptors and membrane fluidity in PC12 cells. J. Biol. Chem..

[133] Szöke E., Borzsei R., Tóth D.M., Lengl O., Helyes Z., Sándor Z., Szolcsányi J. (2010). Effect of lipid raft disruption on TRPV1 receptor activation of trigeminal sensory neurons and transfected cell line. Eur. J. Pharmacol..

[134] Miyake Y., Kozutsumi Y., Nakamura S., Fujita T., Kawasaki T. (1995). Serine palmitoyltransferase is the primary target of a sphingosine-like immunosuppressant, ISP-1/myriocin. Biochem. Biophys. Res. Commun..

[135] Sághy É., Szöke É., Payrits M., Helyes Z., Börzsei R., Erostyák J., Jánosi T.Z., Sétáló G., Szolcsányi J. (2015). Evidence for the role of lipid rafts and sphingomyelin in Ca^2+^-gating of Transient Receptor Potential channels in trigeminal sensory neurons and peripheral nerve terminals. Pharmacol. Res..

[136] Sántha P., Dobos I., Oszlács O., Jancsó G. (2013). Chemical sensitivity of rat primary sensory neurons is regulated by glucosylceramide synthase. Proceedings of the Pain in Europe VIII.

[137] Storti B., Di Rienzo C., Cardarelli F., Bizzarri R., Beltram F. (2015). Unveiling TRPV1spatio-temporal organization in live cell membranes. PLoS ONE.

[138] Sathianathan V., Avelino A., Charrua A., Santha P., Matesz K., Cruz F., Nagy I. (2003). Insulin induces cobalt uptake in a subpopulation of rat cultured primary sensory neurons. Eur. J. Neurosci..

[139] Van Buren J.J., Bhat S., Rotello R., Pauza M.E., Premkumar L.S. (2005). Sensitization and translocation of TRPV1 by insulin and IGF-I. Mol. Pain.

[140] Zhang X., Huang J., McNaughton P.A. (2005). NGF rapidly increases membrane expression of TRPV1 heat-gated ion channels. EMBO J..

[141] Ikonen E., Vainio S. (2005). Lipid microdomains and insulin resistance: Is there a connection?. Sci. STKE.

[142] Biedi C., Panetta D., Segat D., Cordera R., Maggi D. (2003). Specificity of insulin-like growth factor I and insulin on Shc phosphorylation and Grb2 recruitment in caveolae. Endocrinology.

[143] Gustavsson J., Parpal S., Karlsson M., Ramsing C., Thorn H., Borg M., Lindroth M., Peterson K.H., Magnusson K.E., Strålfors P. (1999). Localization of the insulin receptor in caveolae of adipocyte plasma membrane. FASEB J..

[144] Tian Q., Hu J., Xie C., Mei K., Pham C., Mo X., Hepp R., Soares S., Nothias F., Wang Y. (2019). Recovery from tachyphylaxis of TRPV1 coincides with recycling to the surface membrane. Proc. Natl. Acad. Sci. USA.

